# Supplementary figures and images for: Characterization of a novel cell wall-associated nucleotidase of Enterococcus faecalis that degrades extracellular c-di-AMP
Source: PLoS Pathog. 2026 May 20;22(5):e1014206. doi: 10.1371/journal.ppat.1014206 (PMC13229355; doi:10.1371/journal.ppat.1014206)

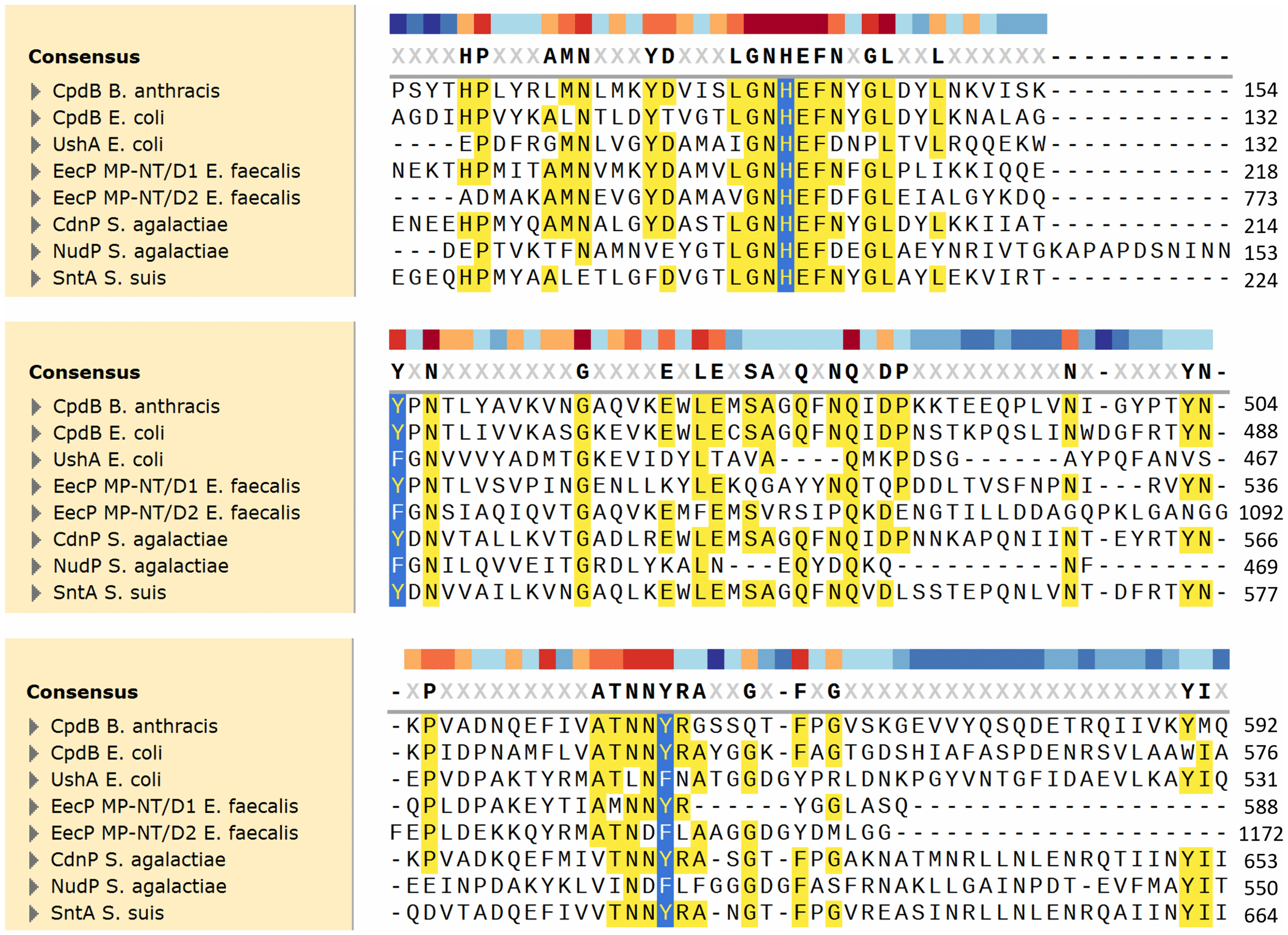

Supplement: S1 Fig — Alignment was performed with Multiple Sequence Comparison by Log-Expectation (MUSCLE) via SnapGene. Sequence sections represented contain conserved histidyl (H) residues found in metallophosphoesterase (MP) domains and tyrosine (Y) or phenylalanine (F) residues found in nucleotidase (NT) domains, all of which are highlighted in blue. Additional conserved residues are highlighted in yellow. Color blocks at the top represent conservation scores for a given residue, with dark red indicating the highest conservation and dark blue indicating the lowest, as calculated by SnapGene. Protein sequences were obtained from genomes of representative strains B. anthracis (Sterne), E. coli (K12 MG1655), S. agalactiae (NEM316), S. suis (SC19), and E. faecalis (OG1RF) available in the NCBI or BioCyc databases. (TIF) [file ppat.1014206.s002.tif]

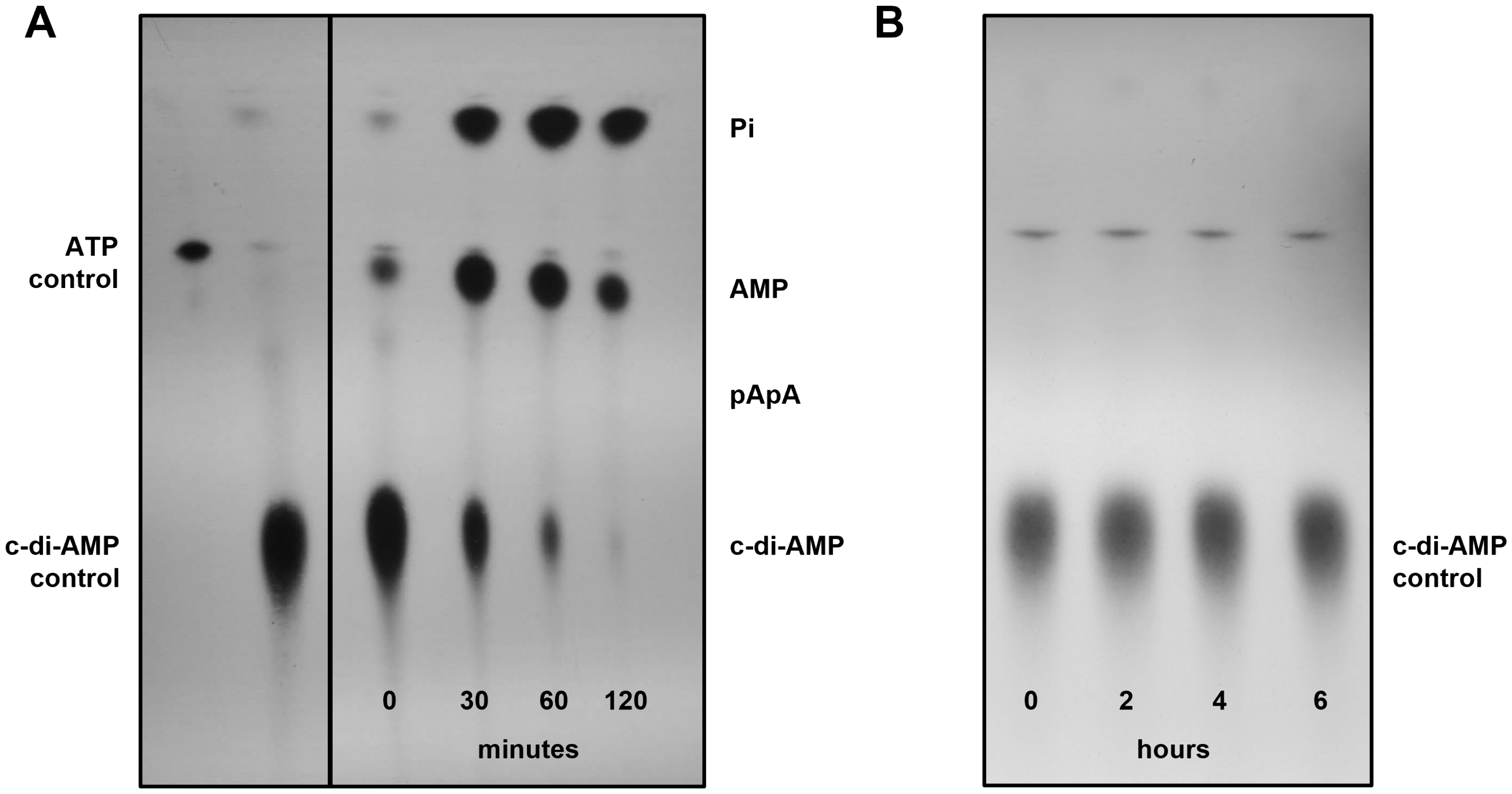

Supplement: S2 Fig — (A) 50 µM of purified rDhhP was mixed with radiolabeled [32P]-c-di-AMP in a 50 mM Tris-HCl, 5 mM MnCl2 buffer. Reaction aliquots were collected at the indicated time points and inactivated by boiling before being resolved via TLC. (B) [32P]- c-di-AMP suspended in reaction buffer was incubated at 37°C for 30 min before inactivation by boiling. The inactivated suspension was then incubated at room temperature and spotted to check for spontaneous degradation for up to 6 h. TLC image is a representative of experiment conducted at least two times. (TIF) [file ppat.1014206.s003.tif]

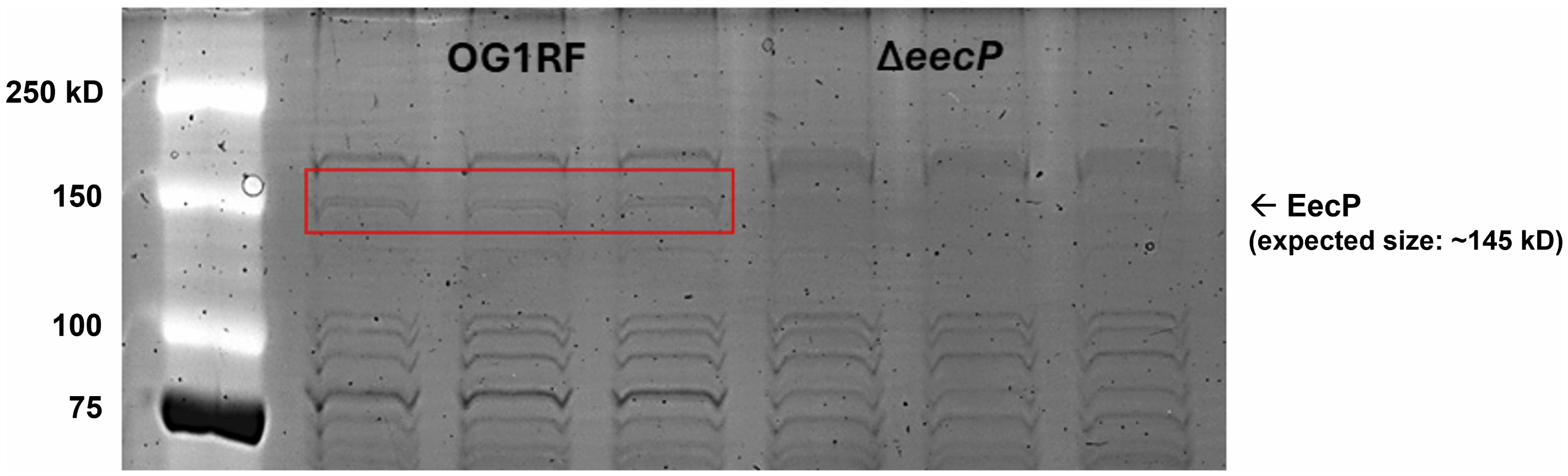

Supplement: S3 Fig — OG1RF and ΔeecP strains were grown to an ~ OD600 0.8, and cell pellets treated with lysozyme (10 mg ml-1) followed by ultracentrifugation to isolate cell wall fractions. Samples were concentrated 100-fold with a 100 kD cut-off Amicon filter and separated by SDS-PAGE. Gels were stained with SYPRO Ruby and visualized with a UV source. The red box highlights the EecP band in OG1RF lanes, which was confirmed by mass spectrometry. Gel image is a representative of experiment conducted at least two times with independent biological replicates. (TIF) [file ppat.1014206.s004.tif]

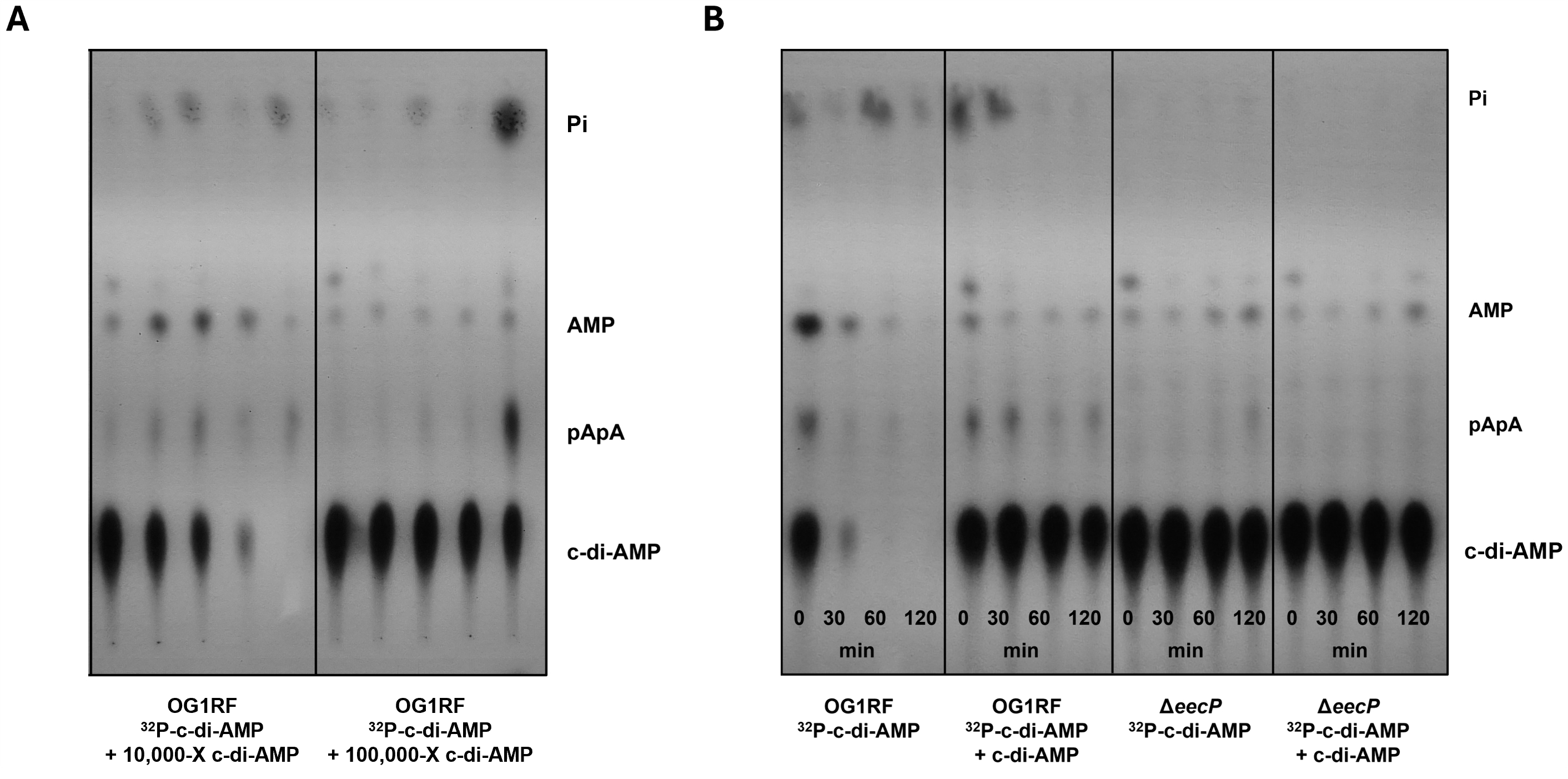

Supplement: S4 Fig — TLC of cell-free supernatants of OG1RF (A-B) or ΔeecP (B) from cultures grown in CDM to ~OD600 0.4, suspended in 50 mM Tris-Cl containing 5 mM MnCl2, and spiked with [32P]- c-di-AMP and cold c-di-AMP (competitor) in 10,000 or 100,000-X excess. Suspensions were sampled over time for c-di-AMP degradation. Reaction aliquots were collected at the indicated time points and inactivated by boiling before spotting on a PEI-cellulose plate for TLC separation. TLC image is a representative of experiment conducted at least two times with independent biological replicates. (TIF) [file ppat.1014206.s005.tif]

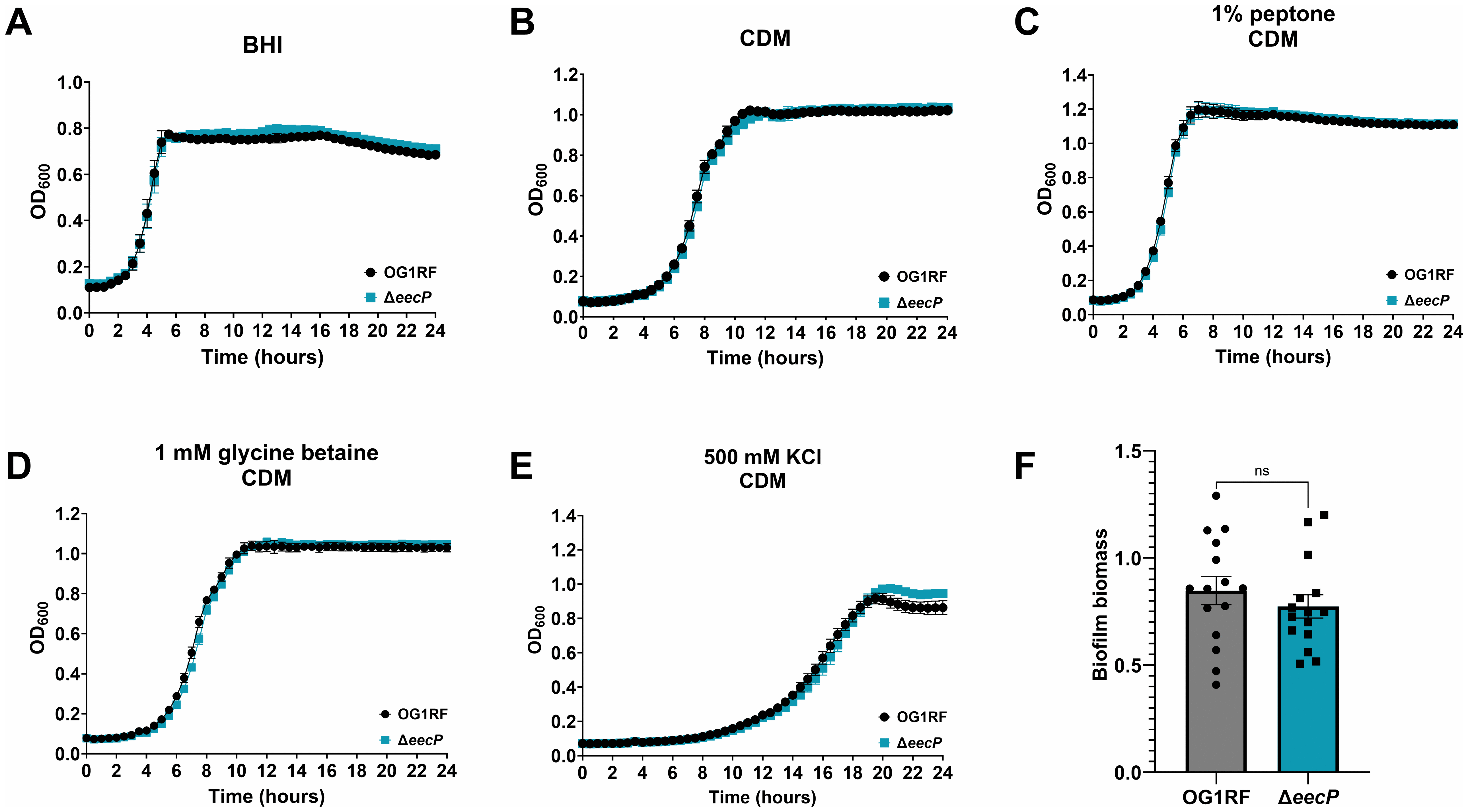

Supplement: S5 Fig — Growth curves of OG1RF and ΔeecP in (A) brain heart infusion (BHI), (B) chemically defined media (CDM), or CDM supplemented with (C) 1% peptone, (D) 1 mM glycine betaine, or (E) 500 mM KCl. Curves represent the average derived from at least three biological replicates. Error bars represent standard deviation. (F) Biofilm biomass quantification of parent strain OG1RF and ΔeecP grown in 96-well plates in BHI for 24 hours. Data points represent fifteen biological replicates from five independent experiments. Unpaired nonparametric t-test was used to determine significance, ****, P ≤ 0.0001, ***, P ≤ 0.001, **, P ≤ 0.01, *, P ≤ 0.05, ns, P ≥ 0.05. Error bars represent the standard error margin. (TIF) [file ppat.1014206.s006.tif]

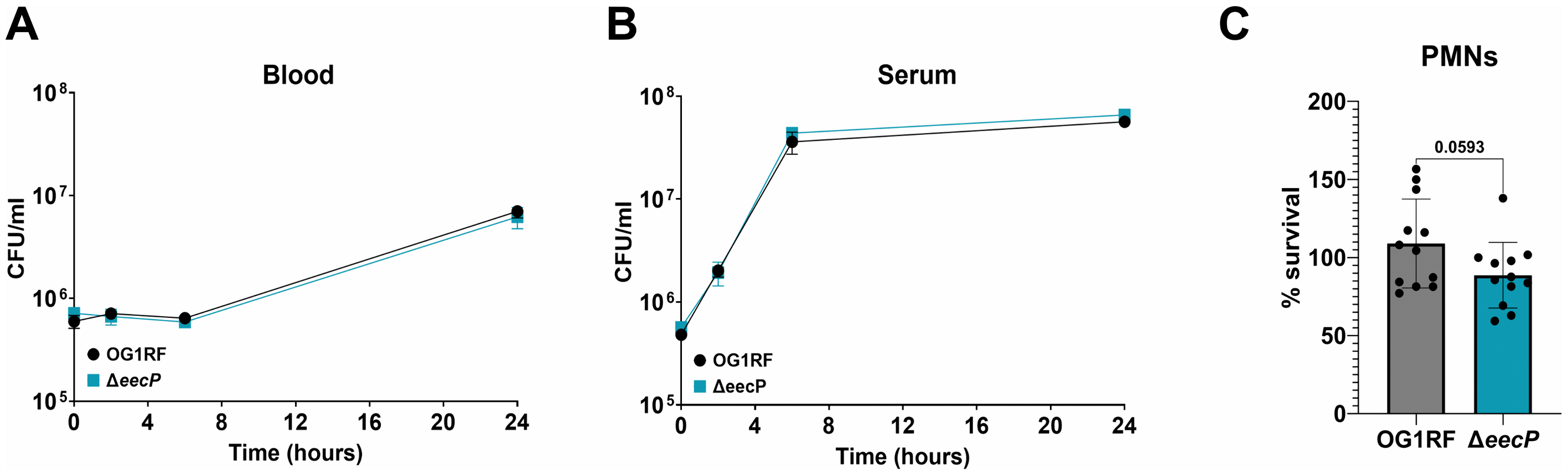

Supplement: S6 Fig — Growth of the parent strain OG1RF and ΔeecP in (A) human blood and (B) human serum. Viability of OG1RF and ΔeecP in (C) polymorphonuclear cells (PMNs). All experiments were conducted using at least three biological replicates. Unpaired nonparametric t-test was used to determine significance, ****, P ≤ 0.0001, ***, P ≤ 0.001, **, P ≤ 0.01, *, P ≤ 0.05, ns, P ≥ 0.05. Error bars represent standard deviation. (TIF) [file ppat.1014206.s007.tif]

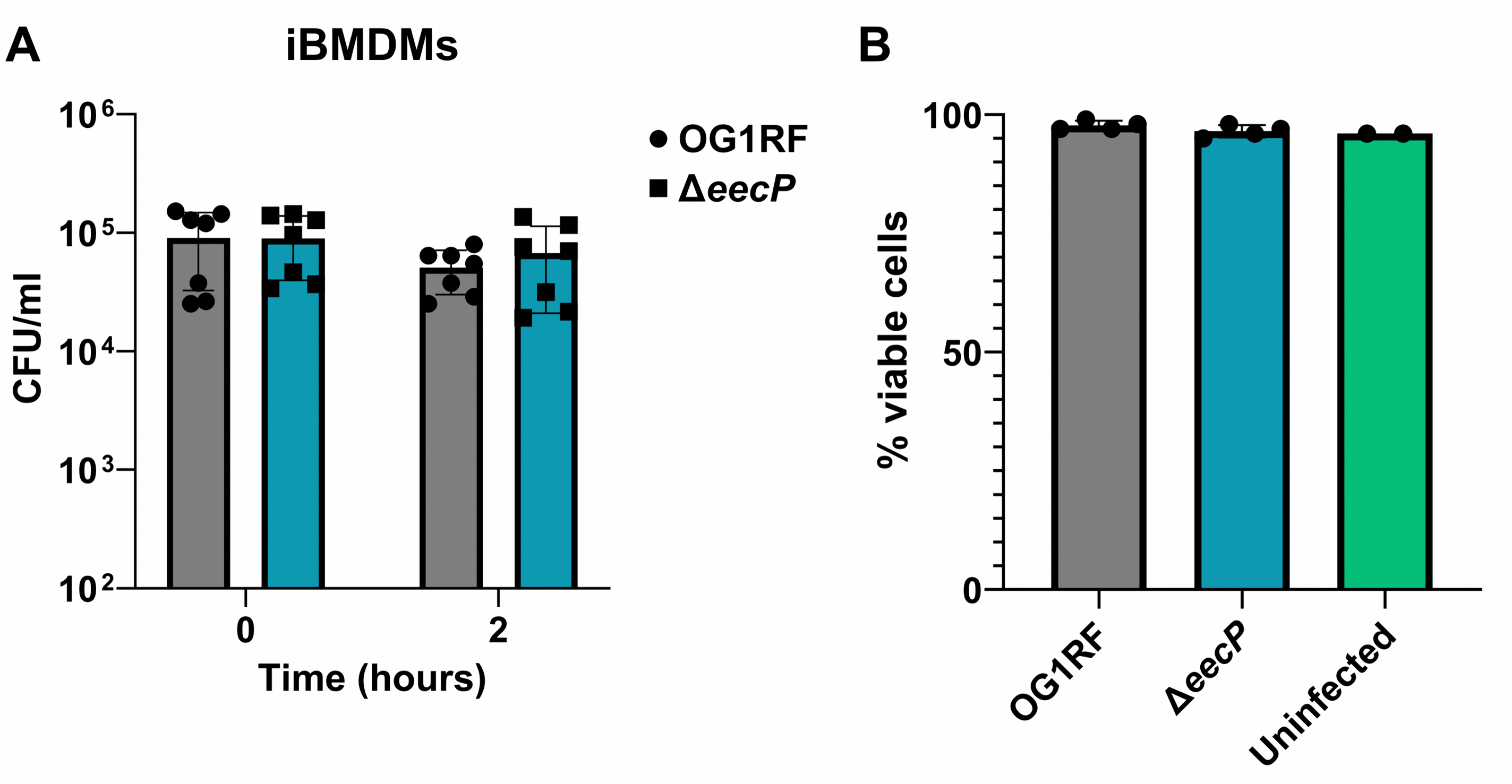

Supplement: S7 Fig — A. Viability of OG1RF and ΔeecP in iBMDMs. B. Viability of iBMDMs 6 h post-infection and antibiotic protection. Macrophage viability was determined with Trypan blue staining and enumerated with a Countess II automated cell counter. Experiments were conducted using at least two biological replicates. Error bars represent standard deviation. (TIF) [file ppat.1014206.s008.tif]

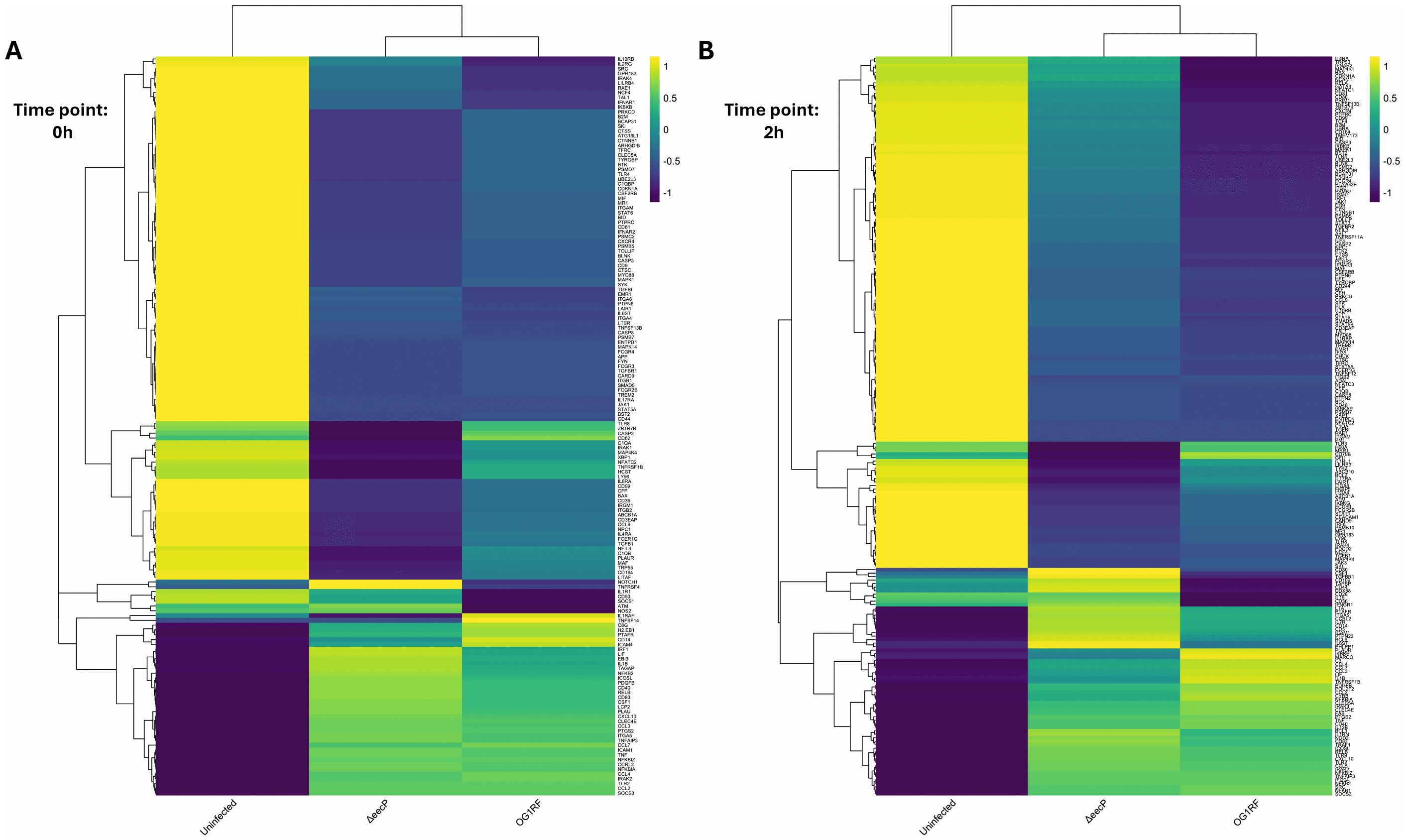

Supplement: S8 Fig — Heatmaps show analytes with significant differences across groups (one-way ANOVA, P ≤ 0.05) at 0 h (A) and 2 h (B). Counts were averaged across biological replicates (n = 4 per group) for visualization and displayed as standardized values (mean = 0, SD = 1). Hierarchical clustering was performed on both rows and columns independently for each time point. (TIF) [file ppat.1014206.s009.tif]
