# Supplementary material for: Characterization of a novel cell wall-associated nucleotidase of Enterococcus faecalis that degrades extracellular c-di-AMP
Source: PLoS Pathog. 2026 May 20;22(5):e1014206. doi: 10.1371/journal.ppat.1014206 (PMC13229355; doi:10.1371/journal.ppat.1014206)
Supplement: Attachment — Submitted filename: Response letter EecP PloS Path Final.docx [file ppat.1014206.s015.docx]

Dear Drs. Wu and Wessels,

Please find enclosed our revised manuscript, "**Characterization of a novel cell wall-associated nucleotidase of *Enterococcus faecalis* that degrades extracellular c-di-AMP**". We would like to thank you for the time and effort devoted to revising our manuscript and for the opportunity to submit this revised version. In particular, we were pleased to learn that the Editor(s) considered the study novel, “addressing an interesting and timely question”, noting that it was “strengthened by additional new data supporting cell-wall association, pharmacologic linkage to cGAS-STING signaling, and expanded immune transcriptional profiling”. However, the Editor(s) also thought that some issues were only partially resolved and that we should either address or discuss these shortcomings. We have carefully revised the manuscript to acknowledge the limitations of certain studies and to provide more cautious interpretations of the EecP substrate specificity and animal studies.

Please find below our point-by-point responses to the comments raised in the prior review.

Major points

1. *Mechanistic linkage to cGAS-STING signaling remains suggestive rather than definitive.*

- We agree that the results are indicative but not fully conclusive, likely reflecting the complexity of c-di-AMP as a host signaling molecule as well as the intricacies of immunological signaling pathways. To address this limitation, we toned down our statements and conclusions in several places.
  - Abstract: “*While a* ΔeecP *strain failed to display relevant phenotypes under most in vitro conditions, it exhibited increased susceptibility to killing by phagocytic cells, a phenotype* ***at least partly associated*** *with cGAS-STING immune signaling*”. (Lines 43-46)
  - Introduction: “…*phenotype that was linked,* ***at least in part****, to c-di-AMP activation of the cGAS-STING signaling pathway*.” (Lines 147-148)
  - “*These results indicate that EecP contributes to E. faecalis survival within phagocytic cells and that the impaired survival of ΔeecP within murine macrophages can be attributed,* ***at least in part****, to activation of the cGAS-STING pathway*” (Lines 419-422)
  - “*This phenotype* ***may be linked*** *with activation of the cGAS-STING signaling pathway, as treatment with either cGAS or STING inhibitors alleviated the virulence attenuation of ΔeecP*” (Lines 599-601).
  - We also added a new sentence in the discussion that reads: “*Follow-up studies using cGAS and STING knockout animals should be conducted to further probe the relationship between the cGAS–STING pathway and ΔeecP phenotypes*” (Lines 601-603).

2. *Substrate specificity remains insufficiently defined in the absence of purified protein, kinetic parameters, or product validation means.*

- As indicated in the first revision, we have not been able to obtain purified EecP for biochemical studies, despite multiple attempts that included heterologous expression (with or without the N-terminal secretion signal and C-terminal LPxTG motifs) and cell-free expression systems.
  - Accordingly, we have clarified in the revised manuscript that the TLC-based assay was employed as an alternative strategy to provide insights into EecP’s substrate specificity (Lines 235-238, 347).
  - To fully acknowledge this limitation, the following sentence was incorporated in the discussion: “*While in our experience EecP was not amenable to large-scale purification using heterologous expression systems in both E. coli and B. subtilis as well as a cell-free expression system, our current understanding would* ***benefit from further characterization using purified EecP***” (Lines 549-552).

3. *In vivo conclusions remain cautious. The manuscript should avoid overinterpreting niche-specific virulence effects and should frame these findings more conservatively as context-dependent infection phenotypes.*

- To be more conservative in the interpretation of our findings, we modified several of our prior statements, including:
  - Abstract: “…*disseminated infection was more severe in mice infected with Δ*eecP, ***suggesting*** *that extracellular c-di-AMP influences infection outcomes, likely through modulation of host immune responses*” (Lines 51-53).
  - Introduction: “…*systemic dissemination was more severe in animals infected with the ΔeecP strain compared to those infected with the parent strain*.” (Lines 155-156)
  - Removed text from discussion suggesting a relationship between c-di-AMP and systemic dissemination. Instead, we have simply stated that the loss of *eecP* led to ***niche-specific phenotypes*** in different mouse models (Lines 520-521).
  - Revised statement from discussion: “*Therefore, additional studies are needed to better understand the relationship between c-di-AMP sensing and E. faecalis colonization of the liver*” (Lines 637-639).
  - Additional revised statement from discussion: “*Despite the impaired bladder colonization of Δ*eecP *in the CAUTI model, both murine models* ***suggest*** *that accumulation of extracellular c-di-AMP may positively impact systemic dissemination*” (Lines 644–646).

Minor issues

1. *Please ensure that the manuscript consistently describes EecP as an immune-modulating factor rather than a straightforward virulence factor.*

- References to EecP as a virulence factor have been replaced by immune-modulating factor or similar (Lines 65-66, 156, and 662-663).

2. *Some biochemical interpretation remains speculative, including the proposed AMP-to-adenosine/Pi activity and the putative role of the second domain; these points should be clearly labeled as hypotheses rather than conclusions.*

- We revised the text throughout the manuscript to indicate that these results are suggestive and will require biochemical validation (Lines 142-143, 360, 363-364, 532-535, 555-556, and 588-592).

3. *The discussion would benefit from a clearer statement of what remains unresolved, particularly regarding export of c-di-AMP, the exact host sensor hierarchy, and whether alternative extracellular nucleotides contribute to the observed immune effects.*

- To address this comment, the following sentences have been added to different parts of the discussion:
  - To acknowledge the possibility of other nucleotides contributing to the observed immune phenotypes: “*Given evidence that EecP may have nucleotide substrates beyond c-di-AMP, additional experiments are required to assess the possible contribution of other nucleotides degraded by EecP to the observed immunological effects*” (Lines 540-543).
  - Regarding identification of c-di-AMP export mechanisms: “*Additionally, our studies indicate that* E. faecalis *can secrete large quantities of c-di-AMP, and thus identification of its c-di-AMP export mechanisms is also warranted*”(Lines 552-554).
  - To address concern regarding host receptors hierarchy: “*While this study supports an association between E. faecalis c-di-AMP and cGAS–STING signaling, future investigations should aim to identify if other host c-di-AMP receptors are more relevant during E. faecalis infections.*” (Lines 626-629).
