# Supplementary material for: Characterization of a novel cell wall-associated nucleotidase of Enterococcus faecalis that degrades extracellular c-di-AMP
Source: PLoS Pathog. 2026 May 20;22(5):e1014206. doi: 10.1371/journal.ppat.1014206 (PMC13229355; doi:10.1371/journal.ppat.1014206)
Supplement: Attachment — Submitted filename: Rebuttal letter EecP PlosPath.docx [file ppat.1014206.s016.docx]

Dear Drs. Wu and Wessels,

Please find enclosed our revised manuscript, "**Characterization of a novel cell wall-associated nucleotidase of *Enterococcus faecalis* that degrades extracellular c-di-AMP**". We would like to thank you and the reviewers for the constructive comments and the time dedicated to revise our manuscript. While recognizing the merit and interest of the study, the Academic Editor and Reviewers requested that we perform few additional experiments to (i) link the cGAS-STING pathway with EecP phenotypes, (ii) assess substrate specificity of EecP, (iii) confirm surface exposure of EecP, and (iv) reevaluate niche-specific virulence claims. We addressed these concerns by conducting several of the suggested experiments and by incorporating new NanoString data that, for the first time, provide a comprehensive “bird’s-eye view” of how elevated c-di-AMP levels globally influence innate immune responses.

Our point-by-point responses to the specific comments made by Academic Editor and Reviewers are delineated below. A marked-up copy with highlighted changes has also been included.

**Editor Comments:**

1) *Link to cGAS–STING signaling. Quantify downstream STING pathway activation during infection with WT, ΔeecP, complemented, and catalytic-His mutant strains (e.g., p-TBK1, p-IRF3, IFN-β mRNA/protein). Establish dependence using cGAS-/- or STING-/- cells (or specific inhibitors) and, where feasible, test at least one host phenotype from Fig. 7D and/or Fig. 8 in cGAS or STING loss-of-function models. Measure extracellular c-di-AMP in the same supernatants/fluids to correlate signal and ligand.*

As requested, we performed experiments to investigate the linkage of elevated extracellular c-di-AMP with the cGAS-STING signaling pathway. In addition to the experiments included in the manuscript, we performed few experiments that were largely inconclusive, possibly due to technical difficulties; therefore, we mention them here briefly. Specifically, we used an immortalized BMDM cell line to assess STING activation via Western blotting. While we were able to detect cGAS, STING, and IRF3 and observed (as expected) no differences in the expression of these constitutive targets in parent or Δ*eecP* mutant-infected iBMDMs, we were unsuccessful in detecting TBK1, pSTING, pTBK1 and pIRF3 phosphorylation after numerous attempts that included technical modifications such as varying antibody concentrations, duration of incubation, etc. These results suggest a limited or transient activation of STING in the conditions tested. As an alternative, we used inhibitors of cGAS (TDI-6570) and STING (H-151) to demonstrate that the increased susceptibility of Δ*eecP* mutant to M1 and M2 macrophages was largely restored upon inhibitor treatment (Fig 7, L407-417 and L591-593). This result indicates that the impaired macrophage survival of Δ*eecP* mutant can be, at least in part, attributed to activation of the cGAS/STING pathway.

To further elucidate the role of c-di-AMP in host sensing of *E. faecalis*, we performed an nCounter Nanostring experiment, which allowed us to probe the expression of 561 immune genes (nCounter Mouse Immunology Panel) in both uninfected and infected iBMDMs. The datasets generated revealed distinct innate immune signatures between OG1RF and Δ*eecP* infections, uncovering differential expression of targets known to be impacted by c-di-AMP, as well as novel targets (Fig 8 and S8 and Tables S1 and S2, L428-457 and L594-619). Due to delays in obtaining certain reagents and the six-month deadline for submitting the revised manuscript, we were unable to quantify c-di-AMP in the corresponding infected cell samples; however, we plan to address this in future studies.

2) *Substrate specificity and products. Because TLC suggests activity on pApA and AMP, provide kinetic parameters (Km, kcat) for c-di-AMP vs. alternative substrates under physiologic conditions and identify products by LC–MS. If EecP is multispecific, temper claims attributing phenotypes solely to c-di-AMP and discuss alternatives; include controls (e.g., apyrase/nucleotidase treatments) to test substrate contribution.*

We failed to mention in our original manuscript that EecP has proven to not be amenable to heterologous expression. Over the lifetime of this project, heterologous expression was attempted in *E. coli* and *B. subtilis* expression systems as well as a cell free expression system (L230-231). The lack of this resource has limited our ability to directly answer valuable questions regarding EecP’s substrate specificity, enzyme kinetics and surface localization. To partially overcome this limitation, we adapted the TLC-based assay that was used to conclusively show that live cells of ePDE+ strains (such as *E. faecalis)* rapidly degrade c-di-AMP into pApA and AMP (Fig 5) to test whether excess unlabeled (cold) nucleotides could stabilize the radiolabeled c-di-AMP signal in the presence of EecP+ cells. This approach revealed that c-di-GMP was an efficient cold competitor while the effect of cold AMP was rather mild. We also found that excess of cold cAMP and cGAMP did not affect [^32^P]-c-di-AMP degradation. We have incorporated these new results in the revised manuscript and have also tempered initial claims that phenotypes of the Δ*eecP* mutant are only linked to extracellular c-di-AMP accumulation (L341-359, L530-533 and L545-563).

3) *Attribution to EecP per se. Confirm surface exposure/cell-wall anchoring (fractionation or protease shaving with controls). Present complementation and catalytic mutant data across key assays, and address possible polar/pleiotropic effects.*

As requested, we isolated cell wall extracts from OG1RF (parent) and Δ*eecP* mutant identifying a ~140 kDa band unique to OG1RF extracts. Mass spectrometry analysis confirmed that the 140 kDa band corresponded to EecP (L258-263).

4) *Niche-specific virulence claims. Re-analyze Figs. 7–8 incorporating the above controls; report effect sizes, randomization/blinding, and power justification, and align conclusions to tissues where effects are (or are not) observed.*

Prior studies and discussions with a statistician helped inform power justification, in which we have determined that ten animals are sufficient to observe differences in virulence. For simplicity, we have revised the animal data to only include 24 h peritonitis infection and 24 h CAUTI infection. Figures 7 and 9 (previously 7 and 8), have been revised to include information on number of animals included. The results are combined from multiple independent experiments. These studies were not randomized or blinded.  We have previously made use of robust nonlinear regression analyses and unpaired t-tests for analysis of these types of data in previous publications. These tests have consistently allowed us to identify trends in the data. In addition, the robust regression and outlier removal (ROUT) test assumes a normal (Gaussian) distribution, we performed normality tests on the data. These tests revealed that the data followed a lognormal distribution. Therefore, to improve stringency, we performed a log transformation of the data to obtain a normal (Gaussian) distribution. These transformed data with normal distribution were then used to perform the outlier tests. This method increased the rigor of our analysis while still accounting for unrepresentative values.

**Reviewers' Comments:**
**Part I - Summary**

**Reviewer #1:** *In the manuscript submitted by Rivera, et al., the authors provided solid evidence that an ectonucleotidase in Enterococcus faecalis, EecP, degrades c-di-AMP and generates Pi. They demonstrated that deletion of eecP elevates extracellular c-di-AMP levels. This mutant also exhibited interesting phenotypes during infection in vitro and in vivo. The role of E. faecalis EecP on bacterial pathogenesis is novel.*

**Reviewer #2:** *In this manuscript, Rivera et al characterize a novel extracellular cell wall anchored phosphodiesterase in Enterococcus faecalis, EecP, which they discovered through in silico analysis. Unique from many previously studied bacterial ePDEs, EecP contains a duplicated MP-NT two-domain structure. The authors found that EecP degrades extracellular cyclic-di-AMP and that this enzymatic activity is dependent on a catalytic histidine residue. Further corroborating this function, they observed increased levels of extracellular cyclic-di-AMP upon deletion of eecP compared to the parental strain. While deletion of eecP did not impact E. faecalis physiology, it did alter E. faecalis virulence—but, interestingly, in some niches but not others (which had similarly been observed by another bacterial ePDE). The data presented are strong and the manuscript is well-written and clear. Mild concerns exist that no experiments were included to 1) evaluate how changes in extracellular cyclic-di-AMP levels between parental and eecP mutant strains alter innate immune signaling, nor to 2) assess contribution of the cGAS/STING pathway to the pathogenesis differences between the parental and eecP mutant strains. Additional minor concerns are listed below.*

**Part II – Major Issues: Key Experiments Required for Acceptance**

**Reviewer #1:** *Apparently, c-di-AMP is not the sole substrate of EecP, as it degrades pApA and AMP as well based on the TLC results. It is known that ectonucleotidases metabolize nucleotides to nucleosides. The substrate of EecP might not limit to c-di-AMP and its intermediates. Since c-di-AMP is not the specific substrate of EecP, the results shown in Figs. 7 and 8 may be related to c-di-AMP, but a consequence due to an effect of EecP on other substrates could not be excluded. Therefore, additional evidence is needed to draw the current conclusions.*

See response to Academic Editor above.

**Reviewer #2:** *The authors suggest a positive correlation between extracellular c-di-AMP levels and systemic dissemination, but do not evaluate host signaling in response to accumulated cyclic-di-AMP nor the dependence of these phenotypes on cGAS/STING signaling. The authors should measure the ability of parental Ef vs eecP mutant strains to promote downstream STING signaling (phosphorylated TBK1 and IRF3, or IFNβ production). Further, dependence of the host-related phenotypes observed in Figures 7D and/or Figure 8 on cGAS/STING signaling should be assessed in cGAS and/or STING KO animals/cells as these are commercially available.*

See response to Academic Editor above.

**Part III – Minor Issues: Editorial and Data Presentation Modifications**
**Reviewer #1:**

1. *L27. “relay signals to stimuli” – relay stimuli to signals?*

Revised text to: “Second messengers are small molecules that relay signals in response to stimuli…” .

2. *L40 and later. EecP is not a phosphodiesterase based on the data presented.*

While we appreciate that only studies using purified enzyme can fully sustain this claim, we argue that is widely accepted to infer gene function based on the presence of highly conserved catalytic domains as well as phenotypic characterization of deletion strains. In addition, our TLC results clearly demonstrate that EecP+ cells rapidly degrade radiolabeled c-di-AMP into pApA and AMP (Figure 5, see also Fig S2 showing c-di-AMP degradation products pApA and AMP using purified intracellular phosphodiesterase DhhP).

3. *Fig. S2. Where is Pi from if the enzyme cleaves one molecule of c-di-AMP into two molecules of AMP?*

We believe that EecP can also degrade AMP but have considerd the possibility that another surface-associated or secreted enzyme is responsible for cleaving AMP into Pi (L529-530).

4. *Fig. 4E. Degradation of c-di-AMP by the ∆eecP mutant, although less dramatic than that by the WT, was not discussed.*

Text discussing that the *eecP* mutant can slowly degrade c-di-AMP appears in multiple sections of the manuscript (L295-298 and L540-544).

**Reviewer #2:**

*- It seems somewhat counterintuitive to label EecP as a virulence factor (lines 138-140) given that deletion of eecP enhances dissemination in both animal models of infection. The authors should clarify this rationale.*

Text has been revised at multiple places to indicate that EecP functions as an immune-modulating factor rather a virulence factor (L52, L64, L151 and L661).

*- Is anything known concerning regulation of eecP (potentially from mining previously published RNA-Seq datasets) as well as the mechanisms by which cyclic-di-AMP is secreted? It would be helpful to add this information to the Introduction and/or Discussion.*

We could not obtain clues on *eecP* regulation or c-di-AMP secretion based on published RNAseq datasets.

*-* *in Fig 1B-C what does the light blue/teal superimposition represent? I did not see these long, disordered regions mentioned in the text. Labeling of residues in these structural models in addition to the color-coding would also be helpful.*

The highlighted residues are described in the figure legend and the teal color described in the text. We have also mentioned the disordered region in the text (L163-169).

*- In Fig 4C-D, there appears to be much higher levels of cyclic-di-AMP in the extracellular compartment compared to the intracellular compartment. Is this the case or is this due to lower amounts of total protein in the supernatant compared to the pellet (the denominator is lower, resulting in a higher normalized value).*

Both intracellular and extracellular c-di-AMP levels were normalized to intracellular protein levels. Thus, it seems that extracellular c-di-AMP do exceed intracellular c-di-AMP levels in *E. faecalis*. This is indeed surprising and we plan to explore this apparently unusual attribute of *E. faecalis* by identifying its c-di-AMP export mechanisms.

*- Figure 6 and possibly Fig 7A-B should be moved to supplementary material.*

Both figures were moved to supplementary material as suggested.

*- eecP not italicized in Fig 7B.*

Corrected.

*- Figure 7D—Is decreased intracellular survival by the eecP mutant here due to decreased initial adherence to or uptake by host cells?*

The macrophage survival experiment was not designed to assess bacterial adherence to or uptake by macrophages. Yet the new data demonstrating robust recovery of the eecP mutant following cGAS or STING inhibition suggest that impaired adherence or uptake is unlikely to account for the observed phenotype. Still, additional experiments would be required to definitively exclude the possibility that reduced adherence or uptake contributes to this effect.

- *More experimental details should be included in the Figure 8 legend—for example, n per group, how many independent animal experiments were performed, are the data shown representative of one experiment or were multiple experiments combined, etc. Animal experiments are known to be variable— can the authors address why outliers were removed in Figure 8? These should be added back in to the results.*

See response to Academic Editor.
